# ErbB Proteins as Molecular Target of Dietary Phytochemicals in Malignant Diseases

**DOI:** 10.1155/2017/1532534

**Published:** 2017-02-13

**Authors:** Alexandru Filippi, Oana-Alina Ciolac, Constanța Ganea, Maria-Magdalena Mocanu

**Affiliations:** Department of Biophysics, Carol Davila University of Medicine and Pharmacy, Bucharest, Romania

## Abstract

ErbB proteins overexpression, in both normal and mutated forms, is associated with invasive forms of cancer prone to metastasis and with stronger antiapoptotic mechanisms and therefore more challenging to treat. Downstream effectors of ErbB receptors mediating these phenotypic traits include MAPK, STAT, and PI3K/AKT/mTOR pathways. Various phytochemical compounds were studied for their large number of biological effects including anticancer activity. Among these compounds, epigallocatechin-3-gallate (EGCG), the main catechin from green tea leaves, and curcumin, component of the curry powder, constituted the object of numerous studies. Both compounds were shown to act directly either on ErbB expression, or on their downstream signaling molecules. In this paper we aim to review the involvement of ErbB proteins in cancer as well as the biologic activity of EGCG and curcumin in ErbB expressing and overexpressing malignancies. The problems arising in the administration of the two compounds due to their reduced bioavailability when orally administered, as well as the progress made in this field, from using novel formulations to improved dosing regimens or improved synthetic analogs, are also discussed.

## 1. Introduction

ErbB proteins are part of the receptor tyrosine kinases family and include four members ErbB1 (also known as EGFR, HER1), ErbB2 (Neu, HER2), ErbB3 (HER3), and ErbB4 (HER4). Since the discovery of the first ErbB protein three decades ago, all four members were shown to be involved in cancer when mutated or overexpressed [1]. ErbB positive cancers are generally associated with a poor prognostic [2, 3], as ErbB overexpression promotes the migration and invasion of cancer cells [4]. Such actions are mediated through the activation of multiple signaling cascades such as MAPK pathway, Akt, and phospholipase C*γ* which lead to the overexpression of several protooncogenic transcription factors [5]. All ErbB receptors can form homo- or heterodimers. Depending on the expression levels, ErbB1 receptors might be present mainly in monomeric form at low expression levels, while ErbB2 receptors are usually found predominantly in aggregates of 3 to 8 molecules [6].

ErbB proteins are used now as molecular markers in therapy, since they can be targeted with either tyrosine kinase inhibitors or with specific monoclonal antibodies, for example, cetuximab and panitumumab against ErbB1 or trastuzumab and pertuzumab against ErbB2 [7, 8]. In spite of the significant success with the use of the monoclonal antibodies in the therapy of ErbB overexpressing cancers, their activity was hindered by the treatment resistance. The resistance to ErbB antibodies may be caused by nuclear localization of ErbB2 [9], miRNA production [10], higher Akt signaling, or steric hindrance by other membrane proteins [11] and raises the need for other therapeutic compounds useful in the treatment of ErbB overexpressing cancers.

Curcumin, a phytochemical constituent of* Curcuma longa*, used in traditional Ayurveda medicine, is now intensively studied due to its anti-inflammatory [12], antitumor [13], and anti-infectious [14] actions. EGCG is the most active and abundant catechin found in* Camellia sinensis* leaves, representing about 16.5% by weight of the water extractable fraction of green tea plants [15]. Among the many actions that EGCG has been shown to exert, are protection against cardiovascular disease [16] and antioxidative [16, 17], antiviral, antibacterial and antifungal [18], and antitumor activities [19]. Both curcumin [20] and EGCG [21] were shown to decrease ErbB2 expression and phosphorylation as well as downstream signaling molecules involved in survival and proliferation of the cancer cells.

## 2. ErbB Proteins and Cancer

ErbB proteins are tyrosine kinase (TK) receptors localized at the plasma membrane level. Up to now, their family includes four members: ErbB1 to ErbB4 and their name was originated from the discovery of avian erythroblastosis viral protein which encoded an abnormal form of epidermal growth factor receptor (EGFR) [5, 22, 23]. A parallel nomenclature for ErbB family frequently used in the scientific field is* human epidermal growth factor receptors*, HER1 to HER4 [24]. Additionally, a mutated form of ErbB2 has been identified in a chemical induced rodent neuroblastoma and the nucleotide sequence able to produce transformation was named* Neu* [25, 26]. However, in human breast or gastric cancer* ErbB2* gene was found rather amplified and not mutated [27–29]. The genes responsible for ErbB family production are part of the four homebox (*Hox*) clusters localized on different chromosomes (Table 1) [7, 30]. The physiological role of ErbB proteins was investigated in several mouse models. The absence of* ErbB1* gene in mouse models was associated with respiratory, gastrointestinal, and skin problems [31–34] and the lack of* ErbB2* gene in mouse models or the therapy against ErbB2 was accompanied by the heart failure [35–37].

Each family member has three main domains: the ligand binding domain, the transmembrane domain, and the kinase domain [38]. The crystal structure of the extracellular/ligand binding domain displayed ligand binding cleft only for three of the family members: ErbB1, ErbB3, and ErbB4, while ErbB2 has no ligand binding cleft [38–42]. For this reason ErbB2 is considered an orphan receptor since it has no ligand and it has a constitutive conformation which was shown to be similar to the activated form of the receptors [8, 43]. The extracellular domain consists of four regions (I to IV) with different functions: I and III are responsible for ligand binding, and region II after ligand binding extends the dimerization arm, while the interaction between region II and IV maintains the extracellular domain in a tethered/inactive form [8].

**Figure 1:**
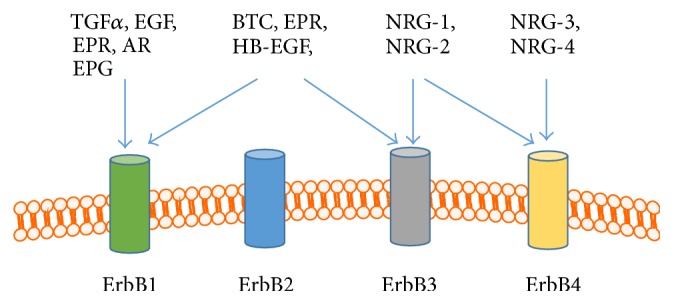
Schematic version of ligand binding for ErbB family. ErbB1 can bind AR, BCT, EGF, TGF*α*, EPG, and EPR; ErbB3 can bind BCT, EPR, HB-EGF, NRG -1, and NRG-2, while ErbB4 can bind all four NRG [5, 7]. AR, amphiregulin; BCT, betacellulin; EGF, epidermal growth factor; EPG, epigen; EPR, epiregulin; BH-EGF, heparin-binding EGF; TGF*α*, transforming growth factor alpha; NRG, neuregulin.

The reported number of ErbB ligands increased to 11, namely, amphiregulin (AR), betacellulin (BCT), epidermal growth factor (EGF), epigen (EPG), epiregulin (EPR), heparin-binding EGF (HB-EGF), transforming growth factor alpha (TGF*α*), neuregulin- (NRG-) 1 to neuregulin-4 [5, 7, 38]. A family of proteases, disintegrin, and metalloproteases (ADAMs) are responsible for generation of ErbB family ligands, which are released by ectodomain shedding from single-pass membrane precursor proteins [44, 45]. The number of amino acid residues responsible for binding ErbB receptors varies from 53 amino acids in case of EGF to 359 amino acids in case of NRG-3 [7]. Up to now, a clear identification of which ligand binds to which ErbB homo- or heterodimer might be a complex task, since 11 ligands can bind 28 possible dimer combinations leading to more than 600 assortments [7]. However, a schematic version of ligand binding to ErbB receptors was depicted in Figure 1 [5, 7]. After the ligand binding, homo- or heterodimerization of ErbB receptors takes place followed by activation of the intracellular kinase domain and by triggering of the numerous intracellular signaling pathways.

Two of the ErbB proteins have incomplete capacity to trigger the intracellular signaling: ErbB2 is an orphan receptor, while ErbB3 has no intrinsic kinase activity. However, the combination ErbB2-ErbB3 was shown to be the most mitogenic one compared to the other dimers of ErbB family [5, 8]. The overexpression of ErbB2 protein in cancer cells made from this member of the family one of the preferred partners for heterodimerization; if there are not enough ErbB family partners on the surface of the cells, ErbB2 can form spontaneously homodimers [5]. Synchronized activation of phosphatidylinositol 3-kinase (PI3K)/Akt pathway, the mitogen-activated protein kinase (MAPK) pathway, phospholipase C*γ* (PLC*γ*), activation of Src kinase, signal transducer, and activator of transcription (STAT) pathway had been associated with increased cell division, survival, migration, differentiation, and inhibition of apoptosis [5]. Furthermore, cross-talking between ErbB signaling pathways and the estrogen receptors (ER), vascular endothelial growth factor (VEGF), insulin growth factor receptor (IGFR), and integrins pathways has been reported [46–50].

At the surface of the cancer cells, ErbB proteins are regularly overexpressed: at about two millions of EGFR in A-431 human epidermoid carcinoma cells and 10^5^-10^6^ ErbB2 proteins in SK-BR-3 breast cancer cell line [51, 52]. The overexpression of ErbB proteins in different cancer types: lung (overexpression of EGFR in 60% of non-small-cell lung cancer, NSCLC), breast (overexpression of ErbB2 in 20–30% of breast cancer), gastric (overexpression of ErbB2 in 22% of gastric cancer), or colorectal cancer (overexpression of EGFR in 25 to 82% of colorectal cancer) made them an attractive therapeutic target [27, 53–56].

The first molecules known to inhibit the activity of ErbB proteins (mainly EGFR and ErbB2) have been the antibodies, followed by small inhibitors of the TK domains [38]. One of the antibodies used to target EGFR had been the following: Erbitux (cetuximab) with antiproliferative activity and the ability to block the cell cycle in G_1_ phase; until now Erbitux has been used in the therapy of colorectal cancer (CRC) and clinical trials are in progress for pancreatic cancer, head and neck squamous cell carcinoma, or NSCLC [8, 57]. Matuzumab and panitumumab are two other monoclonal antibodies which can block EGFR activity; the antibodies are able to inhibit ligand-mediated activation followed by reduced EGFR signaling, cell cycle arrest, and reduced angiogenesis; clinical trials are in progress for CRC and NSCLC [8, 58, 59]. The activation of ErbB2 oncoprotein can be blocked by two monoclonal antibodies: trastuzumab (Herceptin) and pertuzumab (Omnitarg). The first one is able to bind to the IVth region of the extracellular domain of the protein and to trigger antibody-dependent cellular cytotoxicity (ADCC) by attracting the immune cells to the tumor site, while the second one recognizes the dimerization arm of ErbB2 protein being able to block the dimerization process [5, 7, 8, 38]. Pertuzumab in combination with trastuzumab and docetaxel had the approval of the U.S. Food and Drug Administration (FDA) for the treatment of metastatic breast cancer [60] and for the use as neoadjuvant therapy in case of the patients with HER-2 positive breast cancer [61]. Another type of molecules able to block the activity of ErbB proteins is tyrosine kinase inhibitors (TKI): gefitinib (Iressa), erlotinib (Tarceva), and lapatinib; the first two target the tyrosine kinase domain of EGFR with activating mutation (L747-S752 deletion) and are approved for clinical applications in NSCLC [62–64]. A dual inhibitor of both EGFR and ErbB2 tyrosine kinases activity, lapatinib, had been applied mainly in metastatic breast cancer, since in other types of cancer it did not act as an effective inhibitor of the phosphorylation [65, 66].

The monoclonal antibodies and tyrosine kinase molecules showed improved response compared to nonspecific chemotherapy, but the positive responses have been limited to several months, being followed by diseases relapse. The causes of acquired resistance to synthesized molecules are various, for example, the acquisition of a new mutation in the molecular target (T790M EGFR) [67], masking the extracellular epitope of ErbB2 by hyaluronic acid [68], alternative pathways to support proliferation of the cancer cells (IGFR expression) [69], or loss of one of the negative regulators of PI3K/Akt survival pathway (phosphatase and tensin homolog, PTEN) [70].

Since the malignant transformation is not reduced to only one type of event, a complex approach which involves synergic activity of several compounds might bring effective results. The administration of pertuzumab, trastuzumab, and docetaxel to the patients with metastatic breast cancer positive for HER-2 improved progression-free survival [71]. Moreover, with the aim of reducing the tumor size before the surgery, the neoadjuvant chemotherapy was administrated to the patients in early stages of breast cancer positive for HER-2 [72]. Commonly, the adjuvant therapies are accompanied by severe side effects (nausea, vomiting, myelosuppression, cardiotoxicity, etc.) and by multidrug resistance [72]. In the last time, more attention has been paid to the combination between anticancer drugs and natural compounds as potential therapeutic approaches [73]. Some of the advantages of using the natural compounds are related to their pleiotropic effects and to the ability of being well tolerated by the human body [74]. However, most of the studies regarding the administration of natural compounds have been restricted to their preventive effects in cancer and clinical studies to support their therapeutic activity are missing [74, 75]. A summary of the natural molecules known to inhibit the activity of ErbB proteins is presented in Table 2.

## 3. Biological Properties of Dietary Phytochemicals

Over the years dietary phytochemicals were proved to have numerous properties in chemoprevention of chronic diseases, including cancer [74]. For a better understanding about the beneficial properties of the dietary phytochemicals, many experiments,* in vivo* and* in vitro*, have been conducted. Dietary phytochemicals cover a large number of molecules and some were already shown to have biological activity in animal experiments and clinical trials. Their ability to interfere with abnormal proliferation of the cells, such as cancer progression, made them an attractive subject of study [74]. However, if natural compounds found in plants are not expected to treat cancer by themselves, a better understanding of their mechanisms of action can help us adopt the best strategy in cancer prevention or management. Phytochemicals have been classified according to their biological function, chemical structures, or the signaling pathway through which they act; regarding their chemical structure, phytochemicals are classified into polyphenols, alkaloids, carotenoids, and nitrogen compounds [76]. Polyphenols represent the substantial group of phytochemicals which showed many beneficial properties: antioxidant, anti-inflammatory, antitumor, neuroprotective, and antimicrobial [75, 77, 78].

Clinical studies have shown increasing incidence in metastatic cancer due to overexpression of ErbB2 protein [27]. Moreover, the overexpression of ErbB proteins, mutations, or amplification of their genes has been associated with cancer progression [7, 8]. Hence, potential dietary phytochemicals which downregulate ErbB proteins along with inhibiting the tyrosine kinase activity have been investigated.

### 3.1. EGCG

EGCG represents the most abundant polyphenolic catechin found in green tea [79]. Green tea, a popular beverage all over the world, possesses antioxidant properties, due, especially, to its high content in catechins. EGCG structure included eight hydroxyl groups and it was considered to possess anticarcinogenic effect due to its ability to induce apoptosis and to inhibit cell proliferation. The anticancer effect of EGCG was demonstrated through its ability to interfere with cell signaling pathways, NF-kB pathway, and cyclooxygenase-2 (COX-2) involved in cell transformation [80].

The anticarcinogenic properties of EGCG have been observed in multiple* in vitro* and* in vivo* studies. EGCG was proved to inhibit the tumor growth and the activation of ErbB1, ErbB2, and ErbB3 which are expressed in many different human cancer lines. Also, EGCG plays an important role in apoptosis and cell cycle arrest in tumor cells and in inhibiting the signaling pathways (MAPK, PI3K/AKT) and NF-kB activity [81]. Guo et al. demonstrated the effect of EGCG on NF639 cell line, which was obtained from a transgenic mouse, also called virus-HER-2/neu mouse. In this experiment EGCG inhibited the growth of mammary tumor cells in a dose-dependent manner; namely, 65% of the cells were inhibited by 40 *μ*g/mL EGCG and 95% at 80 *μ*g/mL EGCG. However, even after high doses of EGCG, 5% of colony formation showed resistance [82]. EGCG has been shown to have various anticancer effects in A431 epidermoid carcinoma cell line by blocking and inhibiting the tyrosine kinase activity of ErbB1 [83]. The activity of ErbB family members was decreased by EGCG in colon cancer cell lines, breast cancer, and human head and neck squamous cell carcinoma [81]. In the HT29 and SW80 colon cancer cell lines the treatments of EGCG also inhibited the ErbB2 and EGFR activity [84, 85]. Another two cell lines, BT-474 human breast cancer and YCU-H891 human head and neck squamous carcinoma cell lines, overexpressing ErbB2 were treated with EGCG to examine the inhibition of cell growth. After 72 h, EGCG showed a greater inhibition of tumor growth in YCU-H891 line than in BT-474 cells. Also, the effect of EGCG on ErbB2 phosphorylation in both cell lines was followed. The levels of phosphorylated ErbB2 (HER-2) were markedly decreased after 24 h at 10 *μ*g/mL EGCG for YCU-H891 and 30 *μ*g/mL EGCG for BT-474 [21]. In another study the inhibitory effect of EGCG on ErbB2 and ErbB3 overexpressing breast cancer cells was evaluated. Due to its ability to interfere with tyrosine-phosphorylation of ErbB2 and ErbB3, EGCG further inhibited downstream MAPK cascade, leading to the reduction in tumor growth [77]. In pancreatic cancer cells with ErbB protein expression [86] EGCG inhibited PI3K/Akt/mTOR pathway [87] and downregulated the activity of JAK/STAT3 pathway [88].

### 3.2. Curcumin

Curcumin (diferuloylmethane) is a nonflavonoid polyphenol found in turmeric derived from the roots of* Curcuma longa* plant. The yellow spice is widely used in Indian and in Ayurveda medicine as anti-inflammatory agent [89]. Besides curcumin, turmeric contains another three subclasses like demethoxycurcumin (curcumin II), bisdemethoxycurcumin (curcumin III), and cyclocurcumin [90]. Comparable to vitamin C, curcumin has been shown to be a better antioxidant [91]. Curcumin was first isolated from turmeric in 1815 but there was no report about its therapeutic properties until 1970s. Since then, curcumin has been increasingly studied for its promising effects. Besides its anti-inflammatory, anti-infectious, anticarcinogenic, and antioxidant properties, curcumin has been shown to interact with various extracellular and intracellular molecules involved in cancer progression having inhibitory effects on tumor cells [92–94]. The antiproliferative effects of curcumin manifest through the regulation of growth factors, protein kinases, cell receptors, various oncogenic proteins, and the induction of apoptosis and cell cycle arrest in cancer cells [95].

The anticarcinogenic effects of curcumin were tested in both* in vitro* and* in vivo* studies. Experimental studies with curcumin were carried out in human breast cancer cell lines, which expressed p185^*neu*^, the oncoprotein encoded by *ErbB*2/*Neu* gene.* In vitro* studies showed that p185^*neu*^ autophosphorylation and transphosphorylation were inhibited by curcumin. Moreover, the depletion of p185^*neu*^ with a curcumin IC50 (half maximal inhibitory concentration) of 6.5 *μ*M for the 24 h treatment was detected [96]. In another study using SK-BR-3 human breast cancer cell line and COS7 monkey fibroblasts transiently transfected with ErbB2, curcumin downregulated ErbB2 protein by ubiquitination through chaperone-dependent ubiquitin ligase, carboxyl terminus of HSP70-interacting protein (CHIP) [97]. When the effect of curcumin on the human malignant testicular cell line NTera-2 was studied, the inhibition of ErbB2 expression and the reduced expression of activator protein-2 (AP) transcription factor were seen. Also in this study curcumin inhibited the tumor proliferation and induced apoptosis, through the inactivation of AP-2*γ* [98].

**Figure 2:**
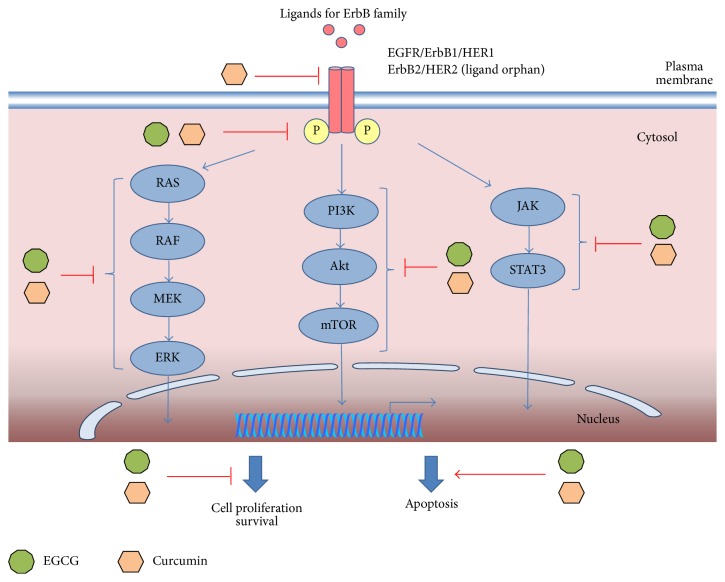
Schematic molecular mechanisms of EGCG and curcumin activity. EGCG was shown to inhibit cell growth in NF639 breast cancer [82], in BT-74 breast cancer, and in YCU-H891 head and neck squamous carcinoma cell lines with ErbB2 overexpression [21]; downregulate the phosphorylation of ErbB proteins in A-4311 epidermoid carcinoma cell line [83], in colon cancer cells, in head and neck squamous cancer cells [81, 84, 85], in BT-74 breast cancer, and in YCU-H891 head and neck squamous carcinoma cell lines [21]; and reduce the activity of downstream MAPK cascade [77], PI3K/Akt/mTOR pathway in pancreatic cancer cells [86, 87], and JAK/STAT3 pathway in pancreatic cancer cells with ErbB expression [88]. In cancer cells with ErbB protein expression, curcumin was able to inhibit the phosphorylation of ErbB2 in breast cancer cells [96], downregulate ErbB2 by ubiquitination in SK-BR3 breast cancer cells and COS-7 fibroblast transfected with ErbB2 [97], inhibit tumor proliferation and induce apoptosis in Ntera-2 human malignant testicular cell line [98], modulate the activity of STAT [99, 100], and inhibit the phosphorylation of Akt and MAPK in BT-474 and SK-BR-3 breast cancer cell lines [20].

Beside the ability to inhibit the activity of EGFR, curcumin has been demonstrated to have pleiotropic biological effects which consist in the ability to modulate various signaling molecules like apoptotic proteins, proinflammatory transcription factors, such as NF-kB, cyclooxygenase-2, AP1, and signal transducer and activator of transcription (STAT) proteins [99, 100]. Additionally, in SK-BR-3 and BT-474 breast cancer cell lines with ErbB3 overexpression curcumin inhibited the phosphorylation of Akt and MAPK pathways [20]. A summary of the mechanisms of action in case of EGCG and curcumin in preclinical studies is illustrated in Figure 2.

Curcumin has also been studied in animal ErbB expressing or overexpressing cancer models where it was very well tolerated and showed promising effects such as tumor size reduction, apoptosis inducement, and tumor prevention [101–103]. Some of those studies are summarized in Table 3.

## 4. Clinical Studies Involving EGCG and Curcumin

Both EGCG and curcumin were studied in a number of phase I and II clinical studies involving patients with developed cancers or at high risk of developing malignancies. Those studies focused on establishing the best treatment regime for further studies and due to the small number of patients employed do not provide much information on the treatment efficacy.

EGCG studies recommended doses of up to 800 mg oral EGCG daily. In those studies EGCG was administered in green tea catechins mixtures, as it was shown that such compositions potentiate the effect of the compound. EGCG was well tolerated in phase I and II clinical studies conducted with the most observed adverse effect being nausea found almost in all studies [104–108]. The largest study conducted included 1075 postmenopausal women at risk of breast cancer randomized in treatment (843 ± 44 mg EGCG daily) and placebo groups and was designed to establish if the toxicity of EGCG was low enough to justify its use as a chemoprevention compound. Due to the fact that percentage of adverse effects in the treatment group did not significantly vary from the one in the placebo group and those adverse effects were mild, the study concludes that “green tea and concentrated catechin extracts have potential for use as a safe, natural supplement for breast cancer risk reduction in healthy populations” [108].

Phase I clinical studies recommended oral doses of about 4 g curcumin, daily [109, 110] or 8 g curcumin daily [111], doses that were well tolerated, with only low levels of toxicity reflected in serum enzyme increase [109] and mild hematological toxicity such as neutropenia [111–113] and diarrhea [109, 110, 113]. In phase II clinical trials curcumin was also well tolerated when administered in combination with standard chemotherapeutic compounds such as docetaxel in advanced metastatic breast cancer [113] or gemcitabine in patients with advanced pancreatic cancer [112]. One study showed the colorectal cancer preventive action of curcumin, as the compound managed to reduce by 40% the number of aberrant crypt foci in high risk patients [110]. In summary, the main effects of EGCG and curcumin in clinical trials are presented in Table 4.

## 5. Bioavailability

### 5.1. EGCG Bioavailability

While epidemiological studies showed positive effects of green and black tea consumption such as reduced ovarian [114], biliary tract [115], or hepatocellular carcinoma [116] cancer risk, researchers working with EGCG have been puzzled by the lack of overlapping between* in vivo* achievable EGCG concentrations and the EGCG concentrations proved efficient in* in vitro* studies. Thus, in the past years there has been an increasing interest in finding EGCG formulations to increase the bioavailability of the compound (Table 5).

*Absorption.* EGCG absorption from decaffeinated green tea administered orally is only about 0.1%–0,15% in rats [117, 118] and about 12–26% in mice [119]. However, the EGCG absorption was shown to be modulated by the coadministration of EGCG with other dietary compounds such as piperine [120], sucrose and ascorbic acid [121], and genistein [122].

Area under the curve (AUC), an area of a concentration-time plot showing when the drug start to be administrated till its concentration in plasma is very low, was used to compare the effect of EGCG under different applications. When administered intragastrically in mice, 6 g/kg piperine increased AUC of total EGCG (coadministered at 75 g/kg) 1.2-fold and unconjugated EGCG 1.3-fold. The putative mechanism for this action was based on higher EGCG absorption through the inhibition of the intestinal glucuronidation of EGCG and, possibly, on gastrointestinal transit slowing [120]. The higher EGCG plasma bioavailability observed in sucrose and ascorbic acid formulation administered to rats may be caused by a longer gastric retention in the case of the higher viscosity formulation and a protective effect of ascorbic acid on EGCG oxidative degradation in the gastrointestinal tract [121].

Genistein was also found to increase EGCG bioavailability (about 1,5-fold increase in AUC) and plasma half-life; however 0.01% EGCG in drinking fluids and 0.2% genistein in food supplementation led to higher tumorigenesis in male adenomatous polyposis coli APC^min/+^ mice, limiting the use* in vivo* of such combinations [122]. EGCG was also shown to modulate its own bioavailability. When low doses, similar to those in green tea, were administered, a second dose of EGCG at 6 hours increases the EGCG available in blood and organs 4–6-fold [123]. On the other hand, AUC was 1.3-fold lower in pretreated mice (3.2 mg/g EGCG for two weeks before the main dose) compared to nontreated mice which receive only the main dose (750 mg/kg EGCG) [124], showing a different modulation mechanism for higher EGCG doses after different term exposure to this flavonoid. 

*Metabolism.* The EGCG low bioavailability is also regulated by multidrug resistance proteins (MRP) and high catechol-O-methyltransferase (COMT) methylation [125] ratios that lead to less active forms [118] like 4′′-O-methyl EGCG, the main methylation product [126, 127]. Also, after absorption, EGCG shows pronounced plasma glucuronidation but, if administered intravenously (i.v.), most of EGCG found in tissues is unconjugated [119]. Glucuronidation occurs in a lesser extent in intestine and liver microsomes, about 12% in 3 h [128]. 

*Distribution.* A study using [^3^H]-labeled EGCG showed wide distribution of the catechin in mouse tissues over a period of 24 hours after EGCG administration for one time, with concentrations in the liver, lung, pancreas, ovary, and mammary gland in the same order of magnitude as those found in plasma; the concentrations in the gastrointestinal tract were the highest, one or two orders of magnitude higher than those found in plasma, depending on the time-point analyzed [123]. 

*Elimination.* EGCG elimination occurs mainly through bile, as shown by a 4-fold or 2-fold higher AUC in the intestine compared to the kidney if decaffeinated green tea extract was administrated i.v. in rats [117] or mice, respectively [119].

In humans, EGCG reaches maximum plasma concentration after 1.3–1.6 hours at lower doses [129] and can reach up to 4 hours for doses of 800 mg [130]. EGCG has an elimination half-life of about 3.4 hours, being cleared completely from plasma after 24 hours [129, 130]. Similar to what was shown in animal studies, in healthy human subjects, EGCG bioavailability can be increased by other phytochemical compounds. EGCG formulated with low caffeine concentrations (EGCG to CAF ratio of 2 : 4) presented an AUC in blood 50% higher than EGCG alone, but higher caffeine concentrations failed to prove the same effect [131].

In conclusion, the bioavailability of EGCG may be moderately increased by other different phytochemicals and by a proper dosing regimen.

### 5.2. Curcumin Bioavailability

Curcumin can be safely administered to humans in doses up to 12 g [132] and one dose escalation study recommended a dose of 6 g curcumin daily for one week, every three weeks [113]. Yet even when a 12 g dose was administered, curcumin serum levels only reached a maximum less of than 60 ng/mL at the 2-hour time-point [132].

*Absorption.* The main reason for the low bioavailability of curcumin is its insufficient water solubility, causing low absorption rates. Thus, a nanoparticle formulation strategy using particles formed from either low molecular weight (5–15 kD) or high molecular weight (50–75 kD) polylactic-co-glycolic acid found no differences between the two polymer sizes but a bioavailability 40 times greater than for free curcumin administration in rats [133].

Another method of increasing curcumin water solubility, and therefore its bioavailability, is complexing curcumin with phosphatidylcholine (PC). Thus, curcumin-PC complexes reach more than triple plasma concentrations and higher AUC at same concentrations than curcumin when it was administered in rats [134]. Other conjugation strategies have also been employed for increasing curcumin stability and solubility in water, such as combining it with hyaluronic acid [135], polyethylene glycol [136], or dendrimers [137], but have not been tested* in vivo* yet.

Curcumin water solubility was also increased successfully using a microemulsion vehicle containing a surfactant, oil, and cosurfactant and this increase in water solubility translated in an about 22 times higher bioavailability of curcumin in rats [138]. A 13-fold increase in bioavailability was observed also in the case of curcumin formulated as amorphous solid dispersion in a matrix consisting of hydroxypropyl methyl cellulose (HPMC), lecithin, and isomalt [139].

A new strategy to overcome curcumin bioavailability problems is the production of novel curcumin analogs. Such curcumin analogs developed for better water solubility can reach bioavailabilities of up to 60% when orally administered in mice [140]. Some curcumin analogs show improved anticancer effects in silico and* in vitro* [141], while others have already been tested* in vivo* and showed improvement in colorectal cancer [142] and glioma [143] survival or inhibited gastric carcinogenesis in mice. Such strategy may prove to find analogs not only with higher bioavailability, but also with improved biological activity. Different strategies for improving bioavailability can be the nanoformulated curcumin analogs which aim at a more specific distribution in tumor tissue [144]. 

*Metabolism.* Curcumin is conjugated in liver and intestine microsome to curcumin glucuronide and curcumin sulfate; hepatic and intestinal alcohol dehydrogenase reduces curcumin to hexahydrocurcumin [145]. There are differences in rat and human curcumin metabolisation, as intestinal microsomes produced 16 times greater curcumin glucuronide in humans than in rats, but hepatic glucuronidation was 3 times higher in rats [145], imposing a certain degree of caution when extrapolating bioavailability results obtained in rats to humans.

Curcumin bioavailability is further hindered by autoxidation processes at physiologic pH. Thus curcumin oxidation forms a large number of intermediary metabolites such as quinone methide, peroxyl radical, endoperoxide, spiroepoxide, vinylether, and cyclopentadione [146] leading to the formation of bicyclopentadione [147]. 

*Distribution.* Curcumin and curcumin nanoparticles have different tissue distributions when administered intravenously. Curcumin nanoparticles present higher bioavailability than curcumin in liver, spleen, and lung than curcumin, while distribution to the heart, kidney, and brain is not greatly affected by nanoformulation, although slightly increased in brain and kidney [133]. 

*Elimination.* Though not entirely demonstrated, it has been theorized that curcumin conjugates are eliminated mainly through renal clearance [148] although curcumin conjugates were also found in feces [149].

In humans, nanoparticle curcumin formulations have already been tested on healthy volunteers and it was shown that they can reach plasma concentrations four times higher at the same time-point when administered in much lower doses of about 200 mg [150] and show 27-fold higher bioavailability than curcumin powder [151]. Also, similar to EGCG, piperine was also found to increase curcumin bioavailability in humans through the inhibition of curcumin glucuronidation which might prove useful in curcumin-EGCG coadministration formulations [152, 153].  Table 6 shows the bioavailability of curcumin in humans and in animal models.

To conclude, even if orally administrated curcumin shows a very little bioavailability, the bioavailability of the compound can be greatly increased using formulations that compensate for the low water solubility of the product and by using curcumin analogs with not only higher solubility but also higher stability.

## 6. Synergistic Activity of EGCG and Curcumin

Because of natural compounds low bioavailability, there is a need to find synergistic associations that might produce anticancer effects even at lower compound concentrations. Several possible combinations between EGCG or curcumin and anticancer drugs had been proven to potentiate the effects of the anticancer drugs in* in vitro* or* in vivo* studies [154–157]. At the same time the combination of EGCG or curcumin with other natural compounds brought additional information about the ability of natural compounds to decrease cell growth, to induce apoptosis or induce cell cycle arrest [158–160]. However a considerable lack of data was noticed regarding the clinical studies concerning the patients with ErbB positive tumors. Additionally, the lack of direct studies regarding ErbB proteins and synergistic activity of EGCG and curcumin was overcome by summarizing in Table 7 the malignant cell lines used for* in vitro* or* in vivo* experiments introduced in this chapter, according to the tissue origin of the cell line and the expression of ErbB proteins.

### 6.1. Synergistic Activity between Natural Compounds and Anticancer Drugs

EGCG was shown to synergistically interact with other drugs employed in cancer treatment and reduced cell viability, increased apoptosis, or inhibited cell growth had been observed [154–156]. Resistance to chemotherapy is an important factor of negative prognostic in oncology patients and therefore the synergy of the natural compounds may prove to play an important role in the treatment of chemoresistant tumors as they were shown to be effective in cisplatin resistant ovarian cancer cells [154, 155]. There are data suggesting a better potentiation of the activity of the compound by sequential administration. In ovarian cancer cells, the effect of cisplatin or oxaliplatin was synergistically increased by curcumin or EGCG administration only when the natural compound was administered after cisplatin at 4 hours, but not before [154, 155]. Curcumin analogs appear to maintain cisplatin potentiating effect, as proved on a cisplatin resistant carcinoma xenograft model [157].  Table 8 summarizes the synergistic activity of EGCG or curcumin and anticancer drugs both in* in vitro* and* in vivo* experiments in ErbB positive cells lines or xenograft tumors.

### 6.2. Synergistic Activity between Natural Compounds

In EGFR+ esophageal cancer lines curcumin and EGCG also greatly reduced p-Erk1/2 expression and increase caspase-3 level [161]. The combination between EGCG and curcumin administrated to breast cancer stem cell model reduced the number of CD44 positive cells and reduced the tumor-sphere formation reversing the stem cell phenotype [162].* In vitro* studies found synergistic effects for either curcumin or EGCG when administered together with other dietary compounds. EGCG was found to exert synergistic apoptosis inducing effects with compounds such as resveratrol or gamma-tocotrienol in breast cancer cells [159], genistein and quercetin in prostate cancer cells [160], or luteolin in several head and neck cancer cell lines or lung cancer cells [158]. Curcumin was also shown to be potentiated by resveratrol in hepatocellular carcinoma cells [163]. In a xenograft model of non-small-cell lung cancer, 20 mg/kg curcumin and 100 mg/kg EGCG coadministration also reduced tumor size through cyclin D1 and cyclin B1 inhibition which led to cell cycle arrest in G_2_/M phase in [161]. The coadministration EGCG and curcumin significantly reduced tumor size in several xenograft nude mouse models [161, 164, 165]. However, little* in vivo* findings support the clinical use of such combinations yet. The synergy studies between EGCG and curcumin or EGCG/curcumin and other natural compounds have been summarized in Table 9.

A new mechanism described for both EGCG and curcumin which might explain in part the synergistic effects of the two compounds when administered together or with other therapeutic compounds is the modulation of multiple miRNA expression [185, 186]. miRNA are small RNA fragments produced by noncoding DNA that have been found to play roles in many cancer-associated mechanisms such as cell differentiation, cell cycle, drug resistance, and metastasis [187]. EGCG was shown to decrease expression of oncogenic miR92, miR93, and miR106 while increasing tumor suppressors miR-7-1, miR-34a, and miR-99a in neuroblastoma cancer cells and curcumin was shown to increase expression of tumor suppressors miR-22, miR-34a, miR-101, miR-141, miR429, miR-200b, miR-200c while downregulating miR199a, in colorectal cancer cells or pancreatic cancer cells [188, 189].

## 7. Conclusions

Up to date the dietary phytochemicals have been used as chemopreventive agents or as potential therapeutic molecules in cancer models and their efficacy as therapeutic agents is missing, suggesting that the most promising results from* in vitro* or* in vivo* experiment might represent a start point for the future approaches in clinical studies. Moreover, the reduced bioavailability of the phytochemicals may represent future challenges for those working in the field. Better understanding of the molecular mechanisms about dietary phytochemicals in translational studies, from* in vitro* to clinical trials, will help us to identify future potential agents or combination of them for the chemoprevention of cancer.

## Figures and Tables

**Table 1:** Genomic location and protein attributes of ErbB family [7, 30].

ErbB member	Genomic location	Molecular mass (kDa)	Number of amino acids
ErbB1	Chromosome 7	134	1210
ErbB2	Chromosome 17	138	1255
ErbB3	Chromosome 12	148	1342
ErbB4	Chromosome 2	147	1308

**Table 2:** Natural molecules which target ErbB proteins.

Name	Target	Mechanism of action	Clinical applications/ *in vitro* or *in vivo* experiments	References
Epigallocatechin-3-gallate (EGCG)	EGFR ErbB2 ErbB3 ErbB4	Inhibits the binding of EGF to EGFR followed by inhibition of the signaling pathways Alters the lipid organization on the plasma membrane (lipid rafts) Promotes internalization of nonactivated EGFR monomer Reduces phosphorylation of EGFR	Clinical trial HNSCC; *in vitro* experiments: epidermoid carcinoma cell line (A-431)	[84, 85, 166, 167]
Quercetin	ErbB2 ErbB3	Reduces the expression level of ErbB2 and ErbB3 Reduces phosphorylation of ErbB2 and ErbB3	*In vitro* experiments: prostate cancer cell lines	[168, 169]
Curcumin	EGFR ErbB2	Induces degradation of ErbB2 by ubiquitination Induces EGFR degradation	*In vitro* experiments: EGFR in several lung adenocarcinoma cell lines; ErbB2 transfected COS7 cell line	[97, 98, 170]
Resveratrol	ErbB2	Downregulates ErbB2 gene Induces apoptosis	*In vitro* experiments: SK-BR-3 breast cancer cell lines	[171]
Apigenin	ErbB2 ErbB3	Inhibits ErbB2 autophosphorylation and transphosphorylation Depletes ErbB2 by polyubiquitination Inhibits the activity of ErbB2/ErbB3 heterodimer	*In vitro* experiments: BT-474, SK-BR-3 breast cancer cell lines	[172]

HNSCC, squamous cell carcinoma of the head and neck.

**Table 3:** Curcumin in animal studies.

Animal model	Treatment	Main results	Ref
EGFR overexpressing A431 epidermoid carcinoma xenograft in nude mice	0.5 mg curcumin i.p. twice a day and 1 h visible light exposure after treatment	ErbB1 phosphorylation reduction; decreased tumor growth through proliferation reduction by 70% and 4-fold increased apoptosis	[101]
Erlotinib resistant ErbB1 mutant NSCLC xenograft in nude mice	1 g/kg body weight curcumin, oral 10 mg/kg erlotinib	Curcumin reduced ErbB1 expression and induced apoptosis Curcumin alone decreased tumor growth and increased erlotinib effect on tumor growth reduction	[102]
ErbB1 expressing LNCaP xenograft in nude mice	2% curcumin in diet	Curcumin decreased tumor volume, decreased mitosis, increased apoptosis, and inhibited tumor angiogenesis	[103]
ErbB2 overexpressing BT-474 breast cancer xenograft in nude mice	45 mg/kg curcumin i.p. twice per week for 4 consecutive weeks	Curcumin reduced the expression level of ErbB2, p-Akt, p-MAPK, and NF-*κ*B; curcumin decreased tumor volume by 76.7%	[20]

**Table 4:** EGCG and curcumin in clinical trials.

Phase	Treatment	Subjects	Main findings	Adverse effects	Ref

*EGCG*					
II	319.8 ± 47.9 mg EGCG oral daily from double brewed green tea	16 women in complete remission after ovarian cancer	5 women free of recurrence at 18 months Not enough evidence to continue the study	Nausea, abdominal pain, vomiting; all adverse effects were of grade 1	[104]
II	1.3 g green tea polyphenols orally, daily, of which 800 mg EGCG was used until prostatectomy	26 men with prostate cancer	EGCG treatment reduced serum levels of HGF, VEGF, IGF-BP3, IGF-I, and PSA	No adverse effects on liver function were observed	[105]
II	500, 750, and 1000 mg/m^2^ green tea extracts orally three times a day or placebo	41 patients with oral premalignant lesions	Higher clinical response rate and histologic response rate in treatment group than in placebo No statistical difference in oral cancer-free survival between the groups	Insomnia (due to caffeine contained in the formulation) Headache Nausea Diarrhea	[106]
Ib	200, 400, and 600 mg poly E extract (50–75% EGCG) or placebo	44 patients with Barrett esophagus	400 mg and 600 mg treatment resulted in organ accumulation of EGCG in esophageal mucosa No significant changes in histological characteristics between cohorts One placebo subject developed high-grade dysplasia	Abdominal pain/discomfort, diarrhea, loss of energy, nausea, upper respiratory infection, and ALT elevation	[107]

*Curcumin*					
I	0.45 to 3.6 g oral curcumin daily, for 4 months	15 patients with advanced colorectal cancer refractory to treatment	Dose-limiting toxicity was not observed; stable disease after 2 months of treatment (2 patients); significant improvement in quality of life after 1 month of treatment (1 patient)	Serum alkaline phosphatase rise, lactate dehydrogenase rise, mild to acute diarrhea associated with longer administration	[109]
II	8 g oral curcumin daily until disease progression	25 patients with advanced pancreatic adenocarcinoma	One patient stable for > 18 months, another for 8 months, and one patient with a brief, 73% reduction in tumor size	No treatment related toxic effects observed	[173]
II	8 g oral curcumin daily + gemcitabine 1 g/m^2^ i.v. weekly × 3 of 4 wk	17 patients with advanced pancreatic cancer	Out of the 11 evaluable patients, 9% had partial response, 36% had stable disease, and 55% had tumor progression	Abdominal fullness or pain Mild hematological toxicity	[112]
I/II	8 g oral curcumin daily, gemcitabine 1 g/m^2^ i.v. days 1 and 8, S-1 orally for 14 consecutive days every 3 weeks	21 patients with gemcitabine resistant pancreatic cancer	Out of the 18 evaluable patients 28% showed stable disease	Hematological toxicity, fatigue (both probably associated with gemcitabine and not curcumin)	[111]
I	0.5 to 8 g oral curcumin daily + 75 to 100 mg/m^2^ docetaxel i.v. every 3 weeks	14 patients with advanced and metastatic breast cancer	5 patients with partial response to the treatment 3 patients with stable disease	Diarrhea Hematological toxicity (neutropenia and leucopenia)	[113]
IIa	2 g or 4 g oral curcumin, daily for 30 days	41 patients with >8 aberrant crypt foci, smokers	4 g curcumin dose significantly reduced the number of premalignant lesions by 40%	Toxicity (grade 1 to 3) or diarrhea	[110]

**Table 5:** Bioavailability of EGCG.

Species	Route	Dose	Plasma/tissue	*C*_max_	AUC	Ref
Rat	i.v.	10 mg/kg	Plasma	4.7 ± 0.9 *μ*g/mL	143.2 ± 32.1 min·*μ*g/mL	[117]
	i.g.	75 mg/kg	Plasma	19.8 ± 3.5 ng/mL	17.4 ± 7.0 min·*μ*g/mL	
Rat	i.v.	10 mg/kg	Plasma	12269.5 ± 2131.8 *μ*g/L	2772.2 ± 479.9 h·*μ*g/L	[118]
	Oral	100 mg/kg	Plasma	11.0 ± 5.9 *μ*g/L	39.6 ± 14.2 h·*μ*g/L	
Mice	i.v.	21.8 *μ*mol/kg	Plasma	13.6 ± 2.0 *μ*mol/L	38.4 ± 5.6 min·*μ*mol/L	[119]
			Prostate	0.31 ± 0.08 nmol/g	56.1 ± 16.0 min·*μ*mol/L	
			Lung	2.66 ± 1.0 nmol/g	91.0 ± 37.3 min·*μ*mol/L	
			Spleen	0.83 ± 0.22 nmol/g	31.7 ± 9.2 min·*μ*mol/L	
			Liver	3.56 ± 0.8 nmol/g	324.0 ± 79.5 min·*μ*mol/L	
			Kidney	2.12 ± 0.6 nmol/g	55.0 ± 17.0 min·*μ*mol/L	
			Small intestine	2.40 ± 1.1 nmol/g	114.0 ± 51.8 min·*μ*mol/L	
			Colon	1.20 ± 0.3 nmol/g	325.3 ± 88.7 min·*μ*mol/L	
	i.g.	163.8 *μ*mol/kg	Plasma	0.04 ± 0.01 *μ*mol/L	45.6 ± 13.5 min·*μ*mol/L	
Human	Oral	2 mg/kg	Plasma	34.71 _ 22.87 ng/mL	213.7 ± 86.4 h·ng/mL	[129]
Human	Oral	95 mg	Plasma	NA	857 h·ng/mL	[131]
Human	Oral	200 mg	Plasma	73.7 ± 25.3 ng/mL	22.5 ± 7.3 min·mg/mL	[130]
	Oral	400 mg	Plasma	111.8 ± 98.6 ng/mL	35.4 ± 21.5 min·mg/mL	
	Oral	600 mg	Plasma	169.1 ± 139.6 ng/mL	101.9 ± 99.7 min·mg/mL	
	Oral	800 mg	Plasma	438.5 ± 284.4 ng/mL	167.1 ± 57.0 min·mg/mL	

i.v.: intravenous; i.g.: intragastric; *C*_max_, maximum concentration; AUC, area under the curve.

**Table 6:** Bioavailability of curcumin.

Species	Route	Dose	Plasma/tissue	*C*_max_	AUC	Ref
Rat	i.v.	25 mg/kg	Liver	NA	9.06 ± 1.55 min·g/mL	[133]
			Heart	NA	3.03 ± 0.85 min·g/mL	
			Spleen	NA	5.72 ± 1.14 min·g/mL	
			Lung	NA	8.98 ± 1.82 min·g/mL	
			Kidney	NA	12.0 ± 0.88 min·g/mL	
			Brain	NA	4.04 ± 0.22 min·g/mL	
Rat	Oral	50 mg/kg	Plasma	13.0 ± 5.8 ng/mL	51.1 ± 25 min·g/mL	[151]
		300 mg/kg	Plasma	37.4 ± 36.1 ng/mL	134 ± 114 min·g/mL	
Rat	Oral	1 g/kg	Plasma	258.64 ng/mL	2483.32 h·ng/mL	[134]
Human	Oral	30 mg/kg	Plasma	1.8 ± 2.8 ng/mL	4.1 ± 7 h·ng/mL	[151]
Human	Oral	10 g	Plasma	50.5 ng/mL	NA	[132]
		12 g	Plasma	57.6 ng/mL	NA	

i.v.: intravenous; i.g.: intragastric; *C*_max_, maximum concentration; AUC, area under the curve; NA, not applicable.

**Table 7:** Cell lines with ErbB protein expression.

Cell line	Tissue/organ	ErbB proteins	Reference
A2780	Ovarian cancer cell line	EGFR: negative, ErbB2: positive, ErbB3: low positive, ErbB4: positive	[174]
Tu212	Hypopharyngeal cancer cell line	EGFR: positive	[158]
A549	Human lung carcinoma cell line	EGFR: positive	[158]
MDA-MB-231	Human breast cancer cell line	EGFR: positive, ErbB2: negative/positive	[175]
MCF-7	Human breast cancer cell line	EGFR, ErbB2: positive	[175]
DLD-1	Human colon cancer cell line	EGFR, ErbB2: positive	[176]
HT-29	Human colon cancer cell line	EGFR, ErbB2: positive	[176]
HCT 116	Human colon cancer cell line	EGFR, ErbB2: positive	[176]
TE-8	Human esophageal cancer cell line	EGFR: positive	[177]
SKGT-4	Human esophageal cancer cell line	EGFR: positive	[178]
HepG2	Human hepatocarcinoma cell line	EGFR, ErbB2: positive	[179, 180]
CWR22R	Prostate cancer cell line	EGFR, ErbB2: positive	[181]
Y79	Human retinoblastoma cell line	ErbB2: positive	[182]

**Table 8:** The main effects of combination of EGCG or curcumin with anticancer drugs.

Anticancer drug	Biological system/model	Doses	Main results	Ref
*EGCG: in vitro experiments*				
Oxaliplatin, cisplatin	A2780 and A2780R ovarian cancer cell lines parental and cisplatin resistant, respectively	ED_50_, ED_75_, ED_90_ (ED, effective dose)	Increased synergism at ED_50_ after the sequential administration of the phytochemical, at 4 h after oxaliplatin or cisplatin treatment (increased growth inhibitory effects)	[154, 155]
Oxaliplatin, cisplatin	DLD-1, HT-29 human colorectal adenocarcinoma cells	100 *μ*M EGCG 20 *μ*M oxaliplatin or cisplatin	Decreased cell proliferation, induced autophagy	[156]

*Curcumin: in vitro experiments*				
Carboplatin, etoposide, vincristine	Y 79 retinoblastoma cells	5–10 *μ*M curcumin 5–10 *μ*g/mL carboplatin 0.1–5 *μ*g/mL etoposide 0.1–5 nM vincristine	Increased apoptosis Curcumin increased the sensitivity of retinoblastoma cells to carboplatin, etoposide, and vincristine	[183]
Oxaliplatin	A2780 and A2780R ovarian cancer cell lines parental and cisplatin resistant, respectively	ED_50_, ED_75_, ED_90_ (ED, effective dose)	Increased synergism at ED_50_ after the sequential administration of the phytochemical, at 4 h after oxaliplatin (increased growth inhibitory effects)	[154, 155]
Cisplatin	A2780R cisplatin resistant human ovarian cancer cell line	1–10 *μ*M curcumin analogs 10 *μ*g/mL cisplatin	Cotreatment increased cytotoxicity (MTT assay), induced G2/M arrest, increased p21 and p53 levels, decreased Bcl-2 and Bcl-XL levels, increased caspase-9, caspase-3, and caspase-7 and PARP levels, increased apoptosis, and reduced STAT3 level	[157]

*Curcumin: in vivo experiments*				
Cisplatin	Xenograft tumor in nude mice with A2780R cells Cisplatin resistant ovarian cancer cells	100 ppm curcumin analogs in feed 4 mg/kg cisplatin i.p.	Cotreatment reduced the tumor volume, decreased constitutive activation of pSTAT3-Tyr705 and pSTAT3-Ser727, decreased Bcl-2 and Bcl-XL levels, and increased PARP levels	[157]

**Table 9:** The main effects of combination between EGCG and curcumin or other natural compounds.

Natural compound	Biological system/model	Doses	Main results	Ref
*EGCG: in vitro experiments*				
Curcumin	TE-8 and SKGT-7 esophageal cancer cell lines	20–40 *μ*M EGCG 20–40 *μ*M curcumin	Reduced viability and invasion ability, reduced pErk1/2 and COX-2, increased caspase-3 level	[161]
	MDA-MB-231 breast cancer cell line	25 *μ*M EGCG 3 *μ*M curcumin	Increased synergistically the cytotoxicity correlated with G2/M phase arrest	[164]
	MDA-MB-231 and MCF-7 breast cancer cell lines transfected with ErbB2 to mimic breast cancer stem cells	10 *μ*M EGCG 10 *μ*M curcumin	Cotreatment reduced the number of CD44 positive cells, reduced the tumor-sphere formation, and reduced the level of pSTAT3	[162]
Resveratrol, *γ*-tocotrienol	MCF-7 breast cancer cell line	50 *μ*M EGCG 25 *μ*M resveratrol 10 *μ*M *γ*-tocotrienol	Inhibited cell proliferation Additive effect when EGCG was combined with *γ*-tocotrienol in reducing the levels of cyclin D1 and Bcl-2 Increased level of antioxidant enzyme NQO1 when all three phytochemicals were used	[159]
Genistein, quercetin	CWR22Rv1 prostate cancer cells	2.5 *μ*M EGCG 2.5 *μ*M genistein 2.5 *μ*M quercetin	Coadministration of EGCG with genistein or quercetin reduced the cell proliferation and increased cell death compared to each treatment alone (the effects were more pronounced in case of combination of EGCG with quercetin)	[160]
Luteolin	Several human head and neck cancer cells from the primary tumor and their lymph node metastasis (Tu212, Tu686, 686LN, and 886LN) and several lung cancer cell lines (H292, A549, H460, H358, H322, H1299, and Calu-1)	30 *μ*M EGCG 10 *μ*M luteolin	Synergistically increased the level of apoptosis (3–5-fold) compared to the additive level Mitochondrial translocation of p53 after the combined treatment	[158]

*Curcumin: in vitro experiments*				
Resveratrol	Hepa 1–6 murine hepatocarcinoma cell line	2.5–40 *μ*M curcumin 10–160 *μ*M luteolin (fixed ratio 1 : 4)	Synergistic effect of the cotreatment consisted in reduced cell survival. The following apoptosis effects were observed: increased annexin V-propidium iodide positive staining, increased caspase-3 activity, increased the number of the nuclei with apoptotic morphology, increased ROS production	[163]
Silymarin	DLD-1, LoVo, HCT116 human colon cancer cells	0–100 *μ*M curcumin 0–100 *μ*M silymarin	Cotreatment induced: increased antiproliferative effects, increased apoptosis, reduced cell survival	[184]

*EGCG: in vivo experiments*				
Curcumin	Xenograft nude mouse model with SKGT-4 esophageal cancer cells	50 *μ*g/kg EGCG 50 *μ*g/kg curcumin	Reduced tumor size after cotreatment Reduced expression of Ki67, pERK, and cyclooxygenase-2 in immunohistochemistry	[161]
	Xenograft nude mouse model with A549 non-small-cell lung cancer cells (females)	100 mg/kg EGCG 20 mg/kg curcumin	Cotreatment protected the mice against weight loss, reduced the tumor growth, reduced cyclin D1 and B2, and reduced the level of the proliferation marker Ki-67	[165]
	Xenograft nude mouse model with MDA-MB-231 breast cancer cells (females)	25 mg/kg EGCG 25 mg/kg curcumin	Cotreatment decreased the tumor volume (by 49%) and the protein expression level of VEGFR-1 (by 78%), but not the levels of EGFR and Akt	[164]
Luteolin	Xenograft nude mice with Tu212 hypopharyngeal cancer cell line	125 mg/kg EGCG 10 mg/kg luteolin	Synergistically decreased in Ki-67 expression and increased in TUNEL positive cells and inhibition of tumor volume	[158]
